# Implant breakage after shoulder arthroplasty: a systematic review of data from worldwide arthroplasty registries and clinical trials

**DOI:** 10.1186/s12891-023-06922-9

**Published:** 2023-10-11

**Authors:** Martin Liebhauser, Gloria Hohenberger, Birgit Lohberger, Georg Hauer, Amelie Deluca, Patrick Sadoghi

**Affiliations:** 1 Department of Traumatology, Klinikum Klagenfurt am Wörthersee, Klagenfurt, Austria; 2 Department of Traumatology, State Hospital Feldbach, Fürstenfeld, Austria; 3 Department of Orthopedics and Trauma, Medical University of Graz, Graz, Austria; 4 Department of Orthopedic Surgery, SKA Warmbad Villach, Villach, Austria

**Keywords:** Implant breakage, Implant fracture, Shoulder arthroplasty, Total shoulder arthroplasty, Reversed shoulder arthroplasty

## Abstract

**Background:**

Implant breakage after shoulder arthroplasty is a rare complication after aseptic loosening, infection or persistent pain, resulting in malfunction of the components requiring revision surgery. This correlates with a high burden for the patient and increasing costs. Specific data of complication rates and implant breakage are available in detailed arthroplasty registries, but due to the rare occurrence and possibly underestimated value rarely described in published studies. The aim of this systematic review was to point out the frequency of implant breakage after shoulder arthroplasty. We hypothesized that worldwide arthroplasty registry datasets record higher rates of implant breakage than clinical trials.

**Methods:**

PubMed, MEDLINE, EMBASE, CINHAL, and the Cochrane Central Register of Controlled Trials database were utilized for this systematic review using the items “(implant fracture/complication/breakage) OR (glenoid/baseplate complication/breakage) AND (shoulder arthroplasty)” according to the PRISMA guidelines on July 3rd, 2023. Study selection, quality assessment, and data extraction were conducted according to the Cochrane standards. Case reports and experimental studies were excluded to reduce bias. The breakage rate per 100,000 observed component years was used to compare data from national arthroplasty registries and clinical trials, published in peer-reviewed journals. Relevant types of shoulder prosthetics were analyzed and differences in implant breakage were considered.

**Results:**

Data of 5 registries and 15 studies were included. Rates of implant breakage after shoulder arthroplasty were reported with 0.06–0.86% in registries versus 0.01–6.65% in clinical studies. The breakage rate per 100,000 observed component years was 10 in clinical studies and 9 in registries. There was a revision rate of 0.09% for registry data and 0.1% for clinical studies within a 10-year period. The most frequently affected component in connection with implant fracture was the glenoid insert.

**Conclusion:**

Clinical studies revealed a similar incidence of implant failure compared to data of worldwide arthroplasty registries. These complications arise mainly due to breakage of screws and glenospheres and there seems to be a direct correlation to loosening. Periprosthetic joint infection might be associated with loosening of the prosthesis and subsequent material breakage. We believe that this analysis can help physicians to advise patients on potential risks after shoulder arthroplasty.

**Level of evidence:**

III.

## Background

The first shoulder arthroplasty was reported in the late 1800s by Themistocles Gluck [1]. In the 1950s, Charles Neer advanced shoulder prosthetics by using the alloy Vitallium [2]. Initial design errors made the implants inherently stable and highly constraint, which resulted in numerous implant breakages (IB) and component loosening [3]. Further research, by focusing on the anatomy, biomechanics, and the use of different materials like Ti6Al-4 V (titanium-aluminum-vanadium) and CoCrMo (cobalt-chromium-molybdenum), extended the durability of the implants significantly [4, 5].

In general, revision surgeries are part of encountered postoperative complications in shoulder arthroplasty (SA) and range from 4 to 10% after 10 years [6–9]. Detailed information concerning the reason for revision is available in almost every arthroplasty registry [7, 10–12] and includes infection, periprosthetic fractures, dislocation and instability, loosening of implanted components, and various rotator cuff pathologies [8, 13].

Specific reasons for revision surgeries are entitled as “other reasons for revision”, but overall there is no difference in the occurrence of dislocation and “other reasons” (0.8%) [8]. Therefore, there is a need to clarify the reasons for IB in shoulder arthroplasties to reduce the number of affected patients, limit health care costs [14], and the need for revision surgeries [15, 16]. Registries and clinical studies should be analyzed and compared to obtain the most probable and real incidence of various complications, such as IB, even if there are differences between registry data and clinical trials with regard to the admission criteria and the generalizability in relation to the examined population.

The aim of this paper was to critically analyze various registries and clinical studies in order to compare and obtain the most probable and real incidence of various complications, such as IB. We hypothesize that overall, the analyzed registry datasets report higher rates of IB after SA compared to data of clinical studies.

This is the first review including the background of implant breakage after shoulder prosthetics. Investigations of the artificial knee and hip joint have been published before [17, 18].

## Methods

**Fig. 1:**
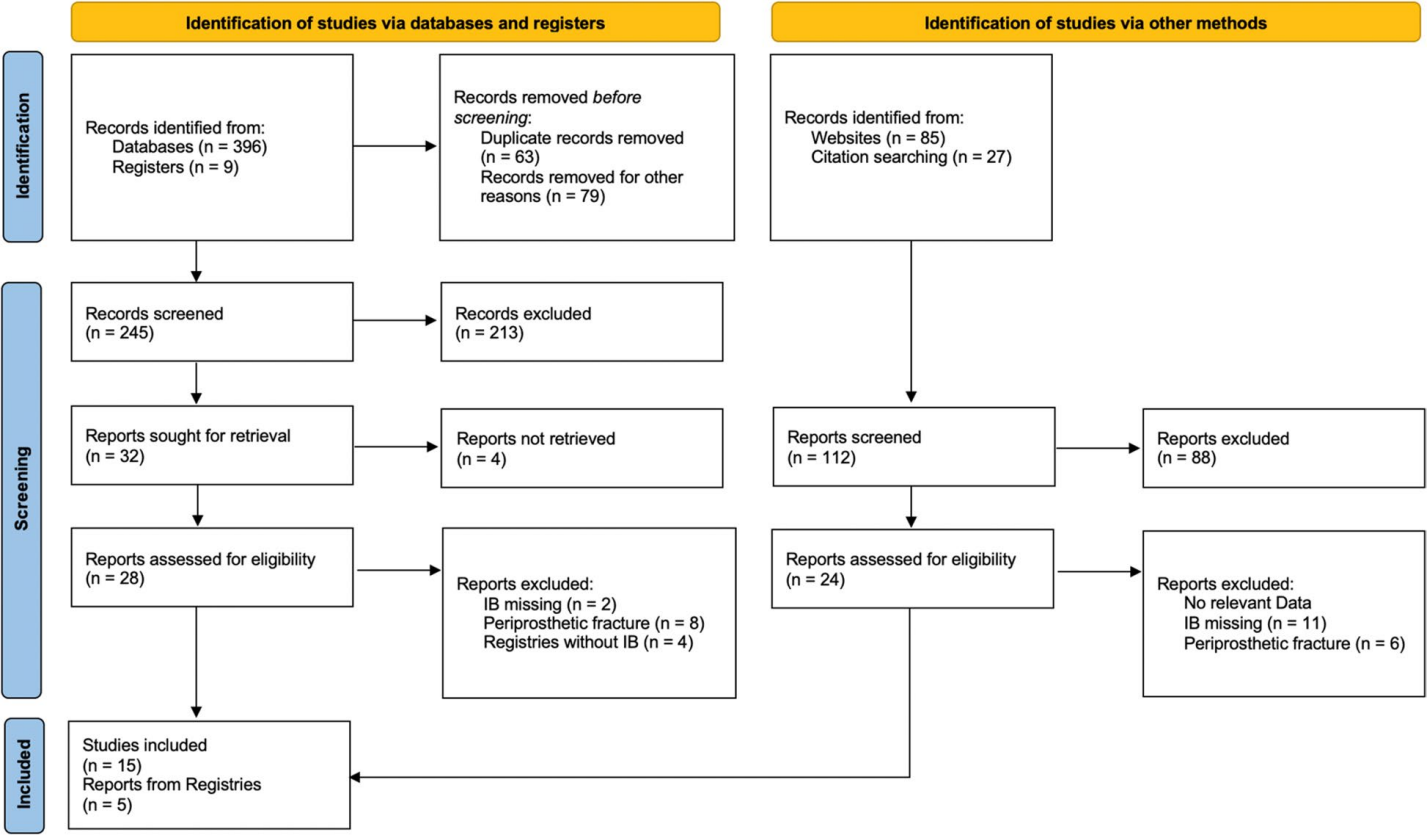
PRISMA 2020 flow diagram for systematic reviews which included searches of databases, registries and other sources. Note: Adopted from Page MJ, et al. (2021) The PRISMA 2020 statement: an updated guideline for reporting systematic reviews. BMJ 2021;372:n71

PubMed, MEDLINE, EMBASE, CINHAL and the Cochrane Central Register of Controlled Trials database were utilized for this systematic review using the items “(implant fracture/complication/breakage) OR (glenoid/baseplate complication/breakage) AND (shoulder arthroplasty)” according to the PRISMA guidelines (Fig. 1) [19]. A reference check of original articles and reviews was done and literature research was performed by reviewing bibliographies and screening peer-reviewed orthopedic journals for relevant articles. Last data search for clinical articles and arthroplasty registers was performed in July 2023. Studies were included in this analysis if (1) the reason for revision was stated within a text or table, (2) the time of observation was given or calculable from the data presented, (3) any kind of IB or fracture was explicitly described. The quality assessment was performed according the Cochrane standards by using the JADAD Score (Table 1) [20]. Experimental studies, case reports, and biomechanical studies were excluded because of heterogeneity of examined specimens or population. Registry data and those from clinical studies consider different populations due to different admission criteria. As a result, there is heterogeneity in the included population, which could be the reason for different results. Large effects are less likely to be fully explained by biases than small effects [21]. Clinically relevant differences are evident in case of a difference of three confidence intervals, which is not the case in the presented work and was published in previous investigations [17, 22]. A general distinction between anatomical, reversed, and subtypes of SA was considered. All described implant fractures, such as within the stem, socket, head, glenoid or baseplate, glenosphere, screws, or polyethylene (PE) inlay, were discussed in this review. PE inlay breakage/damage was included, although similar investigations concerning the knee joint, excluded PE inlay damage as part of the wear and tear mechanism [23]. Clinical studies were included after being reviewed by two independent surgeons (ML and AD) in coherence with the senior author. Furthermore, annual reports from worldwide arthroplasty registries were searched for data containing IB after SA. Detailed information about all listed national registries of SA are summarized in Table 2. The implant breakage per 100,000 observed component years (ocy) was calculated by assuming a linear distribution. The employed formula was introduced by the European Arthroplasty Registry in 2011 [24]. The observed rate/100 component years was equivalent to the yearly revision rate and hence expressed as percentage. The same formula has already been used for answering similar questions regarding the knee or hip joint [18, 22]. Obtained rates were commonly very small and therefore expressed per 100,000 component years, rather than per component itself. The research question was answered by comparing the calculated results.

**Table 1:** Quality assessment of included studies

Study	Jadad scores	Randomization	Double blinding	Drop-out or withdrawals	Allocation concealment
Montoya, F. et al. (2013) [25]	0	H	H	U	H
Somerson, JS. et al. (2018) [26]	0	H	H	U	H
Cil, A. et al. (2010) [27]	0	H	H	U	H
Fucentese, SF. et al. (2010) [28]	0	H	H	U	H
Budge, MD. et al. (2013) [29, 30]	0	H	H	U	H
Martin, SD. et al. (2005) [31]	0	H	H	U	H
Vuillermin, CB. et al. (2015) [32]	0	H	H	U	H
Boileau, P. et al. (2015) [33]	0	H	H	U	H
Styron, JF. et al. (2016) [34]	0	H	H	U	H
Kang, JR. et al. (2019) [35]	0	H	H	U	H
Somerson, JS. et al. (2018) [26]	0	H	H	U	H
Ascione, F. et al. (2018) [36]	0	H	H	U	H
Middernacht, B. et al. (2008) [15]	0	H	H	U	H
Frankle, M. et al. (2005) [37]	0	H	H	U	H
Cappellari, A. et al. (2022) [38]	0	H	H	U	H

L is low risk of bias, H is high risk of bias, U is unclear risk of bias

**Table 2:** Internet presentation of national arthroplasty registries and published articles

Location	Internet site	Publications
Australia	https://aoanjrr.sahmri.com/annual-reports	Graves et al. (2021) [7]
Italy	https://riap.iss.it/riap/en	Romanini et al. (2021) [39]
	http://ior.it/en/curarsi-al-rizzoli/register	Porcellini et al. (2014) [40]
UK	https://reports.njrcentre.org.uk	Leal et al. (2020) [41]
New Zealand	http://nzoa.org.nz	Zhu et al. (2016) [42]
Denmark	http://dssak.ortopaedi.dk	Rasmussen et al. (2019) [43]
Finland	https://thl.fi/en/web/thlfi	
Norway	http://nrlweb.ihelse.net	
Sweden	http://ssas.se	
Netherland	https://www.lroi-report.nl	Aveledo et al. (2019) [44]
USA	https://www.aaos.org	Best et al. (2020) [45]

## Results

Five registries and fifteen clinical studies were included in this review as outlined in the PRISMA 2020 Flow-diagram (Fig. 1). Data were retrieved from the annual reports of Australia, Italy (RIAP and Emilia Romagna), Norway and Denmark [7, 10–12, 46]. Calculations were based on the incidence of revision surgeries after IB. Annual reports from USA, England/Wales/Northern Ireland, New Zealand, Finland, Slovakia, and Canada were evaluated but no relevant data could be found [9, 12, 45, 47–49].

## Clinical studies

Fifteen clinical studies were included and published between 2005 and 2022. Of these, nine studies involved anatomical total shoulder arthroplasty (aTSA) (Table 3) [25–29, 31–34] and six studies included total reversed shoulder arthroplasty (RSA) (Table 4) [15, 26, 35–38]. The study design was carried out retrospectively in all cases except for one article by Budge et al. [29]. It was designed prospectively to evaluate a porous tantalum glenoid component, and was performed by a single surgeon.

**Table 3:** Overview of characteristics of included studies reporting on anatomic total shoulder arthroplasty (aTSA) with implant breakage

Author (year) Reference	Type of SA	Implant Type	Follow up (months)	n (Total)	n (Breakage)	Breakage localization	Fracture rate/100.000 ocy
Montoya (2013) [25]	aTSA	Univers cobalt-chrome metal-backed, bone-ingrowth glenoid component	64	53	5 (9,4%)	Cage screw	1769
Somerson (2018) [26]	aTSA	na	60	1673	5 (0,3%)	na	60
Cil (2010) [27]	aTSA	na	240	1112	2 (0,2%)	Humeral	9
Fucentese (2010) [28]	aTSA	Sulmesh, Zimmer	50	22	3 (13,6%)	Glenoid	3273
Budge (2013) [29]	aTSA	Porous, tantalum-backed glenoid	38	19	4 (21%)	Keel–glenoid face junction	6648
Martin (2005) [31]	aTSA	Plasma-sprayed, screw-fixed uncemented glenoid	90	140	21 (15%)	Glenoid/ Screw	2000
Vuillermin (2015) [32]	aTSA	Modular metal-backed glenoid component TSA (Arthrex, Naples, FL, USA)	66	51	3 (5,9%)	Metal-backed glenoid screw	1070
Boileau (2015) [33]	aTSA	Aequalis MB glenoid prosthesis, Tornier	24	165	6 (3,6%)	Screw	1818
Styron (2016) [34]	aTSA	Trabecular metal anchored glenoid	50	66	1 (1,5%)	na	364

**Table 4:** Overview of characteristics of included studies reporting on reverse shoulder arthroplasty (RSA) with implant breakage

Author (year) Reference	Type	Implant Type	Follow up (months)	n (Total)	n (Breakage)	Breakage localization	Fracture rate/100.000 ocy
Kang, JR. et al. (2019) [35]	RSA	Comprehensive Reverse Shoulder System; Zimmer Biomet, Warsaw, IN, USA)	30	1649	9 (0,5%)	Humeral bearing frctures	218
Somerson, JS. et al. (2018) [26]	RSA	na	60	2390	2 (0,1%)	na	17
Ascione, F. et al. (2018) [36]	RSA	Grammont-style reverse shoulder arthroplasty	98	1035	3 (0,3%)	Diaphyseal/epiphyseal portion	35
Middernacht, B. et al. (2008) [15]	RSA	Delta III TM (DePuy International Ltd, Leeds, UK)	24	479	3 (0,6%)	Fracture of central screw	313
Frankle, M. et al. (2005) [37]	RSA	Lateralised centre of rotation	33	60	5 (8,3%)	glenoid baseplate and screw breakage	3030
Cappellari, A. et al. (2022) [38]	RSA	-	46	91	0	-	0

The overall percentage of IB after SA (aTSA and RSA) in clinical studies ranged between 0.1 and 21.0% (mean 4.1%). There is a higher incidence of IB after aTSA than RSA (1.51 vs 0.3%). A total observation period of 973 months (81 years) was calculated, 682 months (56.8 years) for aTSA and 291 months (24,3 years) for RSA. Cumulative data showed a total of 730,155 ocy. Overall fracture rates per 100,000 ocy diversify between 9 and 6648 (aTSA: 9 to 6648; RSA: 17 to 3030). The mean follow-up time in aTSA was 75 months (38 to 240) and 49 months for RSA (24 to 98).

The most frequent location of IB after TSA was the glenosphere in association with screw breakage in six out of nine listed studies [25, 28, 29, 31–33]. A porus tantalum (PT)—backed glenoid showed 4 fractures out of 19 shoulders at the keel-glenoid face junction. This correlates with the highest incidence out of all studies (21%) and includes a follow-up time of 38 months (range 24–64) [30]. In RSA, the diaphyseal/epiphyseal portion of the hardware was detected in two out of five studies [35, 36], glenoid baseplate and additional screw breakage was described by Frankle et al. [37]. Only one study described just a low number of central screw breakages (0.6%) [15]. The study by Cappellari et al. (2022) described zero IB out of 91 RSA within an observational period of 46 months [38].

## Arthroplasty registries

National, publicly available orthopedic registry data were examined worldwide for the entity of IB after SA. IB itself was only referenced by registries from Australia, Italy (Emilia-Romagna and RIAP), Denmark, and Norway [7, 10, 11, 46] as shown in Table 5. According to the worldwide arthroplasty registries, a total of 101,063 SAs were implanted within 5 to 25 years (1994 – 2021), of which 7,579,725 ocy and 681 cases of IB were identified. Overall, 7.26% of all revision surgeries were due to IB (0.67% of all primary SA). The lowest number of fracture rates of encountered fractures out of all primary SA was found in the Emilia-Romagna Region and RIAP registry in Italy (0.06% in both registries), whereas the highest rates were seen in Norway (0.40%) and Australia (0.88%).

**Table 5:** Revisions due to implant breakage after shoulder arthroplasty reported by the national Arthroplasty Registry of Australia, Italy (RIAP), the Emilia-Romagna Region, Norway, and Denmark

Registry	Published	Data collection	Follow-up (years)	Primary anatomical total shoulder arthroplasties (*N*)	Primary reversed total shoulder arthroplasties (N)	Shoulder arthroplasties TOTAL (N)	Revisions (*N*)	Implant breakage (*N*)	Implant breakage of all revisions (%)	Implant breakage of all primary SA (%)	Fracture rate/100.000 ocy
Australia	2022	1999—2021	22	15,463	42,513	69,243	7104	608	8,56	0,88	40
Italian (RIAP)	2020	2013—2018	5	na	na	1793	45	1	2,22	0,06	11
Emilia-Romagna Region ITALY	2018	2008—2016	8	na	3683	5331	359	3	0,84	0,06	7
Norway	2020	1994—2019	25	na	na	9441	921	38	4,13	0,40	16
Denmark	2020	2004—2019	15	na	na	15,255	954	31	3,25	0,20	14
Total			75			101,063	9383	681	7,26	0,67	9

### The Australian arthroplasty registry

Register data could be integrated into this work from 1 September 1999 to 31 December 2021 (according to the 2022 annual report). A total of 608 IB of aTSA, RSA and subtypes could be identified and are summarized in Table 6. Glenoid erosion and pain were the most common reasons for revision surgery (over 20% respectively), rotator cuff insufficiency, instability/dislocation and loosening exceeded 10% in each case, lysis and infection occurred in less than 5%. IB was mainly accompanied by arthrofibrosis, mispositioning, periprosthetic fracture, and incorrect sizing. SA was divided into subtypes like hemi and total resurfacing, total stemmed, and total reversed. Subsequent delineation was made regarding the location of the IB: Head-, humeral-, glenoid- and glenoid insert component. The most frequently broken component (*n* = 393) was the glenoid insert, followed by the glenoid component (*n* = 146).

**Table 6:**
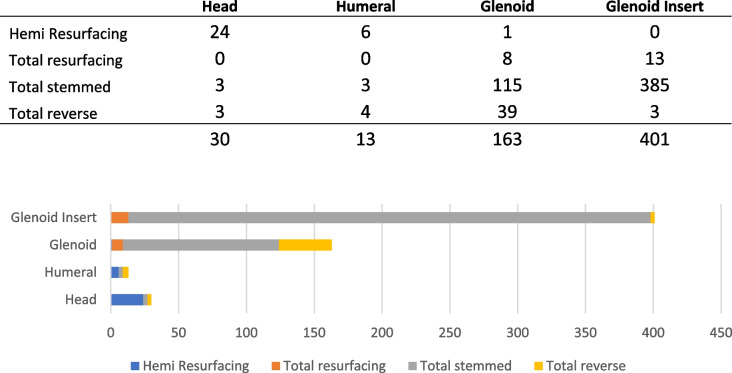
Illustration of the part of the broken implant after failed shoulder arthroplasty and its frequency from 1999 until 2021 out of the Australian Arthroplasty Register (Data from Annual Report 2022)

#### Comparative analysis

Registries and clinical trials include different populations as registries are not affected by the eligibility criteria issues in contrast to clinical studies. Data of clinical trials are less generalizable compared to registry data. Overall, there is population heterogeneity and differences could be solely attributed to that. In analysis of arthroplasty registers clinically relevant and substantial differences are evident, if the confidence interval was exceeded three times, which was already established in previous investigations by Sadoghi and Hauer [17, 18]. If the proposed margin of three confidence intervals was not exceeded, our conclusion does not show a difference with respect to follow up (FU) and IB. Cumulative data for IB of registries and studies are presented in Tables 7 and 8. The total number of observed implants is eleven times higher in registries compared to clinical studies. In national registries, 681 IBs out of 101,063 SAs were observed with a breakage rate per 100,000 implants of 674. In contrast, clinical studies showed 72 IBs out of 9,005 SAs with a breakage rate per 100,000 implants of 800. The ocy in registries was 10 times higher than in clinical studies.

**Table 7:** Overview of the breakage incidence in clinical studies in primary total anatomical shoulder arthroplasty (aTSA) and total reversed shoulder arthroplasty (RSA)

	Primary aTSA [25–28, 33, 45, 47–49]	Primary RSA [15, 29, 31, 32, 34, 45]	Total
Number of shoulder arthroplasties	3301	5704	9005
Number of breakages	50	22	72
Implant breakage (%)	1,5	0,4	0,8
Observed component years	187.607	138.332	325.939
Fracture rate/100.000 ocy	27	16	10

*aTSA* Total anatomical shoulder arthroplasty, *RSA* Total reversed shoulder arthroplasty, *ocy* observed component years

**Table 8:** Data on implant breakages after shoulder arthroplasty from National Arthroplasty registries and clinical trials in comparison

Dataset	Implants (n)	Revisions (n)	Documented implant breakage (n)	Fracture rate per 100,000 implants	Observed component years	Fracture rate per 100,000 observed component years
Clinical studies aTSA	3.301	na	50	1.515	187.596	27
Clinical studies RSA	5.704	na	22	386	138.322	16
**Clinical Studies TOTAL**	**9.005**	**na**	**72**	**800**	**730.155**	**10**
**Registries**	**101.063**	**9.383**	**681**	**674**	**7.579.725**	**9**

*na* not available

## Discussion

The aim of this study was to evaluate the frequency of implant breakage (IB) after shoulder arthroplasty (SA). Even if implant fractures are often seen in connection with aseptic or septic loosening, this could not be proven in the data of the evaluated studies or registers. We found that the incidence of IB in clinical studies and national registry databases is almost equal. The breakage rate per 100,000 observed implants was 674 in various national registries and 800 in clinical studies. The data presented were obtained from the national registry of Australia, Italy, Denmark, and Norway. In this context, the Australian registry data must be underlined separately, as a large part of our data originates from it and significantly contributes to the calculated results. The observation period, the number of shoulder prosthesis and the percentage of implant breakage from studies and registers sometimes show large differences. The calculation method (implant breakage per 100,000 observed component years) is a tool for comparing different data sets, from which clearly comprehensible, almost identical results are shown.

The Australian Joint Registry [7] is updated in autumn every year and includes data on hip, knee, and shoulder arthroplasties in cumulative numbers since 1999. Overall, 68% of all primary SAs and 88% of the included IBs are published in this registry. Revision surgeries were observed more often in patients with a pre-obese metabolic status (32.7%; BMI: 25–29.9). The same cohort presented with the highest number of SAs (35.8%). However, Singh et al. [6], state that there is no correlation between an increased BMI (mean 30, SD 6) or other previous illnesses with an increased ASA score. In contrast, pathologies of the rotator cuff and previous tumor history are mainly responsible for the need of revision surgery with a hazard/risk over 3 times higher than for rheumatoid arthritis [50].

Two studies need to be discussed in detail due to differences in the investigated hardware tools. First, Cil et al. [27] presented a low rate of IB (0.2%) (rate/100,000 ocy = 9) for aTSA, but only the survivorship of the humeral component was observed (implant type was not reported). Second, the prospectively designed study by Budge et al. [29] showed the highest number of fractures, 21% (rate/100,000 ocy = 6648) by using a monoblock porous tantalum glenoid. After receiving the report of the published results, the manufacturing company revised the implants due to the observed high risk of prothesis failure.

Except three clinical studies for aTSA and RSA, all others stated the manufacturing company of the implanted prosthesis. The *Delta Reverse Shoulder System* with its three consecutive versions, was the most used and longest available product for SA. It can be assumed that this is the reason why literature reports the highest rate of complications for this specific prosthetic type. Later, similar complications occurred by using implants from other companies [51].

The type of primary implanted prosthesis depends on several factors, including the biomechanical function of the rotator cuff, the age of the patient, and the extent of the damage to the joint surface [51]. The main distinction in SA is partial or total surface replacement, partial or total anatomical SA or inverse/reversed SA, whereby the anatomy of the joint is changed by lateralization and caudalization of the pivot point and the vector forces. Pure bone-saving prosthesis must be separated from the stem-anchoring cap prosthesis or the inverse shoulder prosthesis. The affected broken components, could be divided according to aTSA and RSA, but only in the Australian arthroplasty registry (Table 8). The use of individual components, to assemble a shoulder prosthesis before implantation, has its advantages and disadvantages. The higher the number of used components, the easier the individual adjustments and, if necessary, the possibility for switching from hemi prosthesis to aTSA or even to RSA is given. A monoblock prosthesis does not offer this option, but it reduces the likelihood of humeral sided complications, like dissociation and component breakage due to a reduced torque stress [52, 53]. In a study by Levy et al. [53], 137 patients who underwent RSA, were retrospectively examined. The minimum follow-up time was 2 years. The study only included patients who were treated with a 2^nd^ generation, lateral-center-of-rotation monoblock RSA. It resulted in an improved range of motion (ROM), a better general health outcome and all PROMs (Simple Shoulder Test, ASES Total, VAS for pain, etc.) were achieved by comparing to preoperative data. Only the internal rotation could not be improved. Instability, loosening, or material fractures were not described. In addition, there was no difference between the outcome of the cemented versus the press-fit technique. A reason for that finding could be, that the two groups (press-fit and cemented) were likely underpowered (116 vs. 21 patients). The expert opinion regarding additional cementation between the bone and glenoid component varies. On the one hand, the additional introduced cement can increase the stability and quality between the components and the bone by filling the trabecular structure; on the other, incorrect cementation with interposition between the back of the component and the bone surface is seen as a risk for implant loosening, fracture and material fatigue [54–56]. A recent manuscript by Kasten et al. (2023) showed no improvement of stability in a biomechanical study, after cementation of the back of the polyethylene glenoid and additional drill holes [57]. Another way to reduce the likelihood of IB or loosening is the "ream and run" technique, which has been described by several authors [58–60]: The humeral component with its artificial head part articulates directly with the glenoid, which is only reamed to achieve a stabilizing concavity to create a maximum glenohumeral contact surface. No intermediate material is implanted. Therefore, no loosening (rocking horse effect) or IB can occur. Several animal studies showed that adequate postoperative exercise leads to a regeneration of the glenoid cartilage. One of the observed procedural disadvantages is, that it is only applicable to selected patients with osteoarthritis, capsulorrhaphy arthropathy and post-traumatic arthritis [58].

### Area of implant breakage

**Fig. 2:**
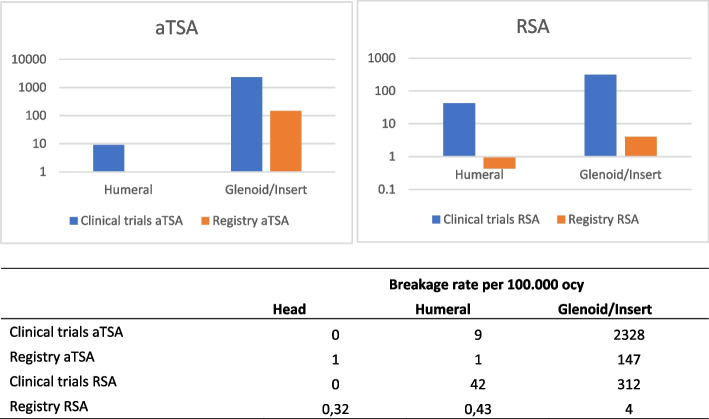
Comparison of breakage rate per 100.000 ocy, data extracted from clinical trials and Australian Registry. Note: Breakage rate per 100.000 observed component years is shown logarithmically. aTSA = anatomical total shoulder arthroplasty; RSA = total reversed shoulder arthroplasty; ocy = observed component years

For illustration, the comparison of calculated breakage rate per ocy (aTSA and RSA) from register and clinical studies concerning breakage location is shown (Fig. 2). The calculated fracture rate per 100,000 ocy is presented logarithmically, breakage of the “head” component was not considered graphically, because values are too low. The amount of IB in this figure is higher in clinical studies than in registries. This results from the sole consideration of aTSA and RSA (the implant fractures after resurfacing, as listed in the Australian registry, were not included).

#### Glenoid/Baseplate

The review by Ravi et. al. (2021), confirms that glenoid (401 out of 3,041; 16%) and baseplate failure (83 out of 3,041; 3%) occur more frequently than pain and stiffness (62 out of 3,041; 2%) [61]. Breakage of the glenoid component can occur on the pegs or keels, baseplate, or screws. Keeled glenosphere baseplates are more difficult to fix to the prepared bone than pegged designs [62, 63]. Surface irregularities, fractures, rim erosions and central wear on PE within the aTSA system can be observed after 2.5 years [64]. PE abrasions stimulate an increase in the local macrophage activity. Due to the formation of a membrane, it further leads to a resorption of the trabecular bone and the bone-cement interface, resulting in loosening of the components [65, 66]. Loosening of the glenoid through snatching and the subsequent breakage of the implant can often be demonstrated in the case of brittle bones or pronounced osteoporosis [16, 67]. It can be further observed if direct contact with the bony glenoid is insufficiently prepared or the glenoid component has been poorly positioned [68] or, if there is a superior bone defect of more than 50 percent [69]. Pure tissue ingrowth glenoid components were already described in the 2000s with high revision rates of up to 12.5%. This has also been appreciated in connection with metal-poly loosening and subsequent screw breakage [64]. At the beginning of the twenty-first century, metal-backed glenoids (MPG) showed a higher rate of loosening than all-PE components [70, 71]. A direct association with material fracture has not been described. Nevertheless, it can be assumed, because IB with component loosening was described in several articles [16, 64, 67, 72]. A review by Kim et al. (2020), however, showed that newer and modern MPGs performed significantly better in terms of loosening, ROM, and clinical scores compared to the conventional older designs [73].

#### Glenosphere

In a retrospective study of 479 RSA, Middernacht et al. [15] described the frequency of signs of loosening in the glenosphere after an observation period of at least 12 months (range 12–72 months). A disengagement of the glenosphere was demonstrated in 16 cases (3.2%), an additional break of the central screw in 3 cases, after 24 months (range 12–48 months). Due to the noticeably poorer clinical outcome, with an average Constant Murley Score of 46 (range 26–61), compared to 62 (range 45–81) for disengagement without IB, an absolute indication for revision surgery was described [15].

#### Cage screw

The screw connection that is used for primary stabilization of the glenoid, breaks in the event of loosening, as can occur by notching after RSA [51, 72, 74]. In a study of 324 patients, Roche et al. presented that notching showed a significantly poorer initial stability of the baseplate, ROM and clinical scores after a minimum follow-up of 5 years [16].

#### Humeral component

According to the Australian registry, IB in the humeral component are the least common (Table 8). Over a period of 6 years, only 13 cases were reported (2.14% of ALL described implant fractures during this period) [7]. The study group by Cil et al. (2010) concur with the results, although the survival rate of the humeral components, after an observation period of 20 years, was calculated by the Kaplan Maier curve and resulted to be 83%. Of the 1.584 examined SAs, only 0.2% showed a breakage of the humeral component, and underwent revision surgery [27]. The literature describes a clear connection between a radiologically confirmed implant loosening and an infection with Cutibacterium acnes, 2–3 years after the primary surgery [75]. According to Middernacht et al. [15], loosening of the stem is also associated with an increased risk of breakage, but no connection between periprosthetic joint infection (PJI) and IB could been seen in the literature so far.

To highlight the incidence of glenosphere disengagement and clinical outcome, Middernacht et al. [15] presented their results in a series of 479 RSAs (468 Delta III and 11 Aequalis) with a minimum observatory time of 12 months. Three percent of RSAs showed a disengagement of the glenosphere (16 of 479). The author described the breakage of the central screw in three cases dur to complete disengagement (Delta III). Partial disengagement was seen in 45.4% of the Aequalis and in 1.7% of the 468 Delta prosthesis. Scarlat et al. [51] identified several complications after RSA after reviewing 240 papers in the timeframe from 1996 to 2012. He described complication rates varying from 10 to 65% in long term series. A direct correlation between loosening/disengagement and the breakage of the screw fixation of the glenosphere in RSA has been reported several times in the literature [18, 23, 37, 51, 72]. However, this could not be proven in the data of the studies or registers.

In comparison to data after knee arthroplasty, it was found, that clinical studies showed an incidence of IB at least twice as high as registry data (Fracture rate/100,000 Implants: 285 versus 129) [23]. The incidence of IB after hip arthroplasty is 304/100,000 Implants in registries, the most affected component is the cup, whereas in clinical studies it is the stem [18].

There are limitations to this study. As with all systematic reviews, the quality of data is dependent on the data of the main source, in that case arthroplasty registers. Only non-randomized studies of interventions (NRSI) could be included in this review because no randomized controlled trials (RCTs) were found that addressed the topic. The total amount of data collected is not precise enough to draw definitive conclusions concerning specific failure mechanisms. A correlation-, sensitivity-, or subgroup analysis could not be performed because of a lack of data. There is a probability, that the incidence of implant fractures is underrepresented, but at least the frequencies as reported.

A recent case report by Ramirez et al. (2020) describes the history of a 63-year-old woman, in which a glenosphere loosening due to central screw breakage, remained undiagnosed two years after primary RSA. The authors firmly believe that this IB was an avoidable complication. The glenosphere stability on the base plate is related to the central screw fixation because there is no morse taper. It is of paramount importance that the screw engages properly to achieve compression and long-term stability [72]. If radiological postoperative imaging appears unremarkable in the control, an additional radiological evaluation can be carried out in the event of persistent symptoms in order to avoid misdiagnosis.

This is the first study to evaluate the incidence of IB after SA by evaluating national registries and clinical trials. Although the observation periods, the number of implanted prostheses, and the specified number of implant fractures were presented differently, a valid tool was used to compare the data sets by calculating the fracture rate per 100,000 ocy. With regard to the hypothesis, we found that the rate in clinical studies and registers revealed no clinically relevant difference (9 vs. 10). The goal should be the accurate collection of data from national registries, modeled on the Australian one. The prospective study of Budge et al. [29], enhances that registry datasets could help to identify implants with a higher rate of failure. Surgeons do have the possibility to inform patients more accurately about potential IB after SA.

## Conclusions

Implant breakage occurs more rarely than aseptic loosening or infection after shoulder arthroplasty. The incidence of implant breakage in registries and clinical studies is almost equal. During follow-up care, a slowly increasing pain must be further clarified, even without primary X-ray findings. Primary loosening might be the main reason of IB, investigations are needed to underline this hypothesis with data. Additional work-up, associating periprosthetic joint infections with loosening of the prosthesis and subsequent material breakage, would be desirable. The authors believe that in clinical practice the result of this analysis can help to advice patients on potential complications following shoulder arthroplasty.

## Data Availability

All data generated or analyzed during this study are included in this published article.

## Abbreviations

SA: Shoulder Arthroplasty
RSA: Reversed Shoulder Arthroplasty
ADL: Activities of daily live
AN: Avascular necrosis
aTSA: Anatomical Total Shoulder Arthroplasty
BMI: Body mass index
COR: Center of Rotation
FU: Follow up
IB: Implant breakage
ISP: Infraspinatus Muscle
MD: Musculus Deltoideus
MPG: Metal-backed glenoid
NRSI: Non-randomized studies of intervention
OA: Osteoarthritis
ocy: Observed component years
PE: Polyethylene
PJI: Periprosthetic joint infection
PROMs: Patient reported outcome measures
RA: Rheumatoid Arthritis
RC: Rotator cuff
RCA: Rotator Cuff Arthropathy
RCT: Randomized controlled trails
ROM: Range of Motion
SSC: Subscapularis Muscle
SSP: Supraspinatus Muscle

## References

[1] Bankes MJ, Emery RJ (1995). Pioneers of shoulder replacement: Themistocles Gluck and Jules Emile Pean. J Shoulder Elbow Surg.

[2] Neer CS 2nd (1955). Articular replacement for the humeral head. J Bone Joint Surg Am.

[3] Fenlin JM (1975). Total glenohumeral joint replacement. Orthop Clin North Am.

[4] Berliner JL (2015). Biomechanics of reverse total shoulder arthroplasty. J Shoulder Elbow Surg.

[5] Mehta N (2020). The Biomaterials of Total Shoulder Arthroplasty: Their Features, Function, and Effect on Outcomes. JBJS Rev.

[6] Singh JA, Sperling JW, Cofield RH (2011). Revision surgery following total shoulder arthroplasty: analysis of 2588 shoulders over three decades (1976 to 2008). J Bone Joint Surg Br.

[7] Graves S, Turner C (2021). Australian Orthopaedic Association National Joint Replacement Registry (AOANJRR). Hip, Knee & Shoulder Arthroplasty: 2021 Annual Report.

[8] Amundsen A (2019). Low revision rate despite poor functional outcome after stemmed hemiarthroplasty for acute proximal humeral fractures: 2,750 cases reported to the Danish Shoulder Arthroplasty Registry. Acta Orthop.

[9] McKie J, Hobbs T, Frampton C, Coleman B, Pattett A. The New Zealand Joint Registry, in Twenty-One Year Report. The New Zealand Joint Registry 2020: 2020. www.nzoa.org.nz/nzoa-joint-registry.

[10] Rasmussen JV, Jakobsen J, Brorson S, Olsen BS. The Danish Shoulder Arthroplasty Registry: clinical outcome and short-term survival of 2,137 primary shoulder replacements. Acta Orthop. 2012;83(2):171–3. 10.3109/17453674.2012.665327 PMC3339532 22329671

[11] Urakcheeva I, Biodi A, Torre M (2019). Italian Arthroplasty Registry. Annual Report 2019 - Addendum.

[12] Havelin LI (1999). The Norwegian Joint Registry. Bull Hosp Jt Dis.

[13] Graves S, Turner C (2019). Australian Orthopaedic Association National Joint Replacement Registry (AOANJRR) Hip, Knee & Shoulder Arthroplasty: 20th Annual Report.

[14] Edwards TB, Morris BJ. Shoulder Arthroplasty E-Book. Elsevier Health Sciences. 2018.

[15] Middernacht B (2008). Glenosphere disengagement: a potentially serious default in reverse shoulder surgery. Clin Orthop Relat Res.

[16] Roche CP (2013). The impact of scapular notching on reverse shoulder glenoid fixation. J Shoulder Elbow Surg.

[17] Hauer G (2020). Survival rate and application number of total hip arthroplasty in patients with femoral neck fracture: an analysis of clinical studies and national arthroplasty registers. J Arthroplasty.

[18] Sadoghi P (2014). The incidence of implant fractures after total hip arthroplasty. Int Orthop.

[19] Page MJ (2021). The PRISMA 2020 statement: an updated guideline for reporting systematic reviews. BMJ.

[20] Jadad AR (1996). Assessing the quality of reports of randomized clinical trials: is blinding necessary?. Control Clin Trials.

[21] Glasziou P (2007). When are randomised trials unnecessary? Picking signal from noise. BMJ.

[22] Sadoghi P (2013). Revision surgery after total joint arthroplasty: a complication-based analysis using worldwide arthroplasty registers. J Arthroplasty.

[23] Gilg MM (2016). The incidence of implant fractures after knee arthroplasty. Knee Surg Sports Traumatol Arthrosc.

[24] Labek G (2011). Quality of Publications regarding the Outcome of Revision Rate after Arthroplasty.

[25] Montoya F (2013). Midterm results of a total shoulder prosthesis fixed with a cementless glenoid component. J Shoulder Elbow Surg.

[26] Somerson JS (2018). Analysis of 4063 complications of shoulder arthroplasty reported to the US Food and Drug Administration from 2012 to 2016. J Shoulder Elbow Surg.

[27] Cil A (2010). Survivorship of the humeral component in shoulder arthroplasty. J Shoulder Elbow Surg.

[28] Fucentese SF (2010). Total shoulder arthroplasty with an uncemented soft-metal-backed glenoid component. J Shoulder Elbow Surg.

[29] Budge MD (2013). Results of total shoulder arthroplasty with a monoblock porous tantalum glenoid component: a prospective minimum 2-year follow-up study. J Shoulder Elbow Surg.

[30] Budge MD (2013). A biomechanical analysis of initial fixation options for porous-tantalum-backed glenoid components. J Shoulder Elbow Surg.

[31] Martin SD, Zurakowski D, Thornhill TS (2005). Uncemented glenoid component in total shoulder arthroplasty. Survivorship and outcomes. J Bone Joint Surg Am.

[32] Vuillermin CB (2015). Catastrophic failure of a low profile metal-backed glenoid component after total shoulder arthroplasty. Int J Shoulder Surg.

[33] Boileau P (2015). Metal-backed glenoid implant with polyethylene insert is not a viable long-term therapeutic option. J Shoulder Elbow Surg.

[34] Styron JF (2016). Survivorship of trabecular metal anchored glenoid total shoulder arthroplasties. Tech Hand Up Extrem Surg.

[35] Kang JR (2019). Primary reverse shoulder arthroplasty using contemporary implants is associated with very low reoperation rates. J Shoulder Elbow Surg.

[36] Ascione F (2018). Long-term humeral complications after Grammont-style reverse shoulder arthroplasty. J Shoulder Elbow Surg.

[37] Frankle M (2005). The Reverse Shoulder Prosthesis for glenohumeral arthritis associated with severe rotator cuff deficiency. A minimum two-year follow-up study of sixty patients. J Bone Joint Surg Am.

[38] Cappellari A (2022). Reverse shoulder arthroplasty for treatment of proximal humerus complex fractures in elderly: A single institution experience. Injury.

[39] Romanini E, Schettini I, Torre M, Venosa M, Tarantino A, Calvisi V, Zanoli G (2021). The rise of registry-based research: a bibliometric analysis. Acta Orthop..

[40] Porcellini G, Combi A, Merolla G, Bordini B, Stea S, Zanoli G, Palandini P (2014). The experience of the RIPO, a shoulder prosthesis registry with 6-year follow-up. Musculoskelet Surg..

[41] Leal J, Murphy J, Garriga C, Delmestri A, Rangan A, Price A, Carr A, Prieto-Alhambra D, Judge A. Costs of joint replacement in osteoarthritis: a study using the National Joint Registry and Clinical Practice Research Datalink datasets. 2020. 10.1002/acr.24470. 10.1002/acr.24470 33002322

[42] Zhu M, Ravi S, Frampton C, Luey C, Young S (2016). New Zealand Joint Registry data underestimates the rate of periprosthetic joint infection. Acta Orthop..

[43] Rasmussen JV, Oslen BS (2019). The Danish Shoulder Arthroplasty Registry. Obere Extremität.

[44] Aveledo R, Holland P, Thomas M, Ashton F, Rangan A. A comparison of the minimum data sets for primary shoulder arthroplasty between national shoulder arthroplasty registries. Is international harmonization feasible? Shoulder Elbow. 2019;11(2 Suppl):48–55. 10.1177/1758573218755569 PMC6688150 31447945

[45] Best MJ (2021). Increasing incidence of primary reverse and anatomic total shoulder arthroplasty in the United States. J Shoulder Elbow Surg.

[46] Bordini B, Stea S, Ancarani C, et. al. REPORT of Regional Register of Orthopaedic Prosthetic Implantology. Bologna: 2018. http://www.ior.it/en/curarsi-al-rizzoli/register-orthopaedic-prosthetic-implants.

[47] Porter M, Howard P (2018). National Joint Registry for England, Wales, Northern Ireland and the Isle of Man - 15th Annual Report.

[48] Hobbs T , Frampton C (2018). The New Zealand Joint Registry.

[49] De Reus IMA, Spekenbrink-Spooren A, et. al. Dutch Arthroplasty Register (LROI). Netherlands Orthopaedic Association (NOV): 2018. www.lroi-report.nl.

[50] Sperling JW, Cofield RH, Rowland CM (1998). Neer hemiarthroplasty and Neer total shoulder arthroplasty in patients fifty years old or less. Long-term results. J Bone Joint Surg Am.

[51] Scarlat MM (2013). Complications with reverse total shoulder arthroplasty and recent evolutions. Int Orthop.

[52] Cuff D (2011). Torsional stability of modular and non-modular reverse shoulder humeral components in a proximal humeral bone loss model. J Shoulder Elbow Surg.

[53] Levy JC (2019). Primary monoblock inset reverse shoulder arthroplasty resulted in decreased pain and improved function. Clin Orthop Relat Res.

[54] Yian EH (2005). Radiographic and computed tomography analysis of cemented pegged polyethylene glenoid components in total shoulder replacement. J Bone Joint Surg Am.

[55] Terrier A, Buchler P, Farron A (2005). Bone-cement interface of the glenoid component: stress analysis for varying cement thickness. Clin Biomech (Bristol, Avon).

[56] Matsen FA (2008). Glenoid component failure in total shoulder arthroplasty. J Bone Joint Surg Am.

[57] Kasten P (2023). Impact of polyethylene glenoid cementation technique on cement mantle integrity and stability after cyclic loading: a computed tomography and biomechanical study. J Shoulder Elbow Surg.

[58] Matsen FA (2015). The ream and run: not for every patient, every surgeon or every problem. Int Orthop.

[59] Somerson JS (2017). Clinical and Radiographic Outcomes of the Ream-and-Run Procedure for Primary Glenohumeral Arthritis. J Bone Joint Surg Am.

[60] Chawla SS (2021). Drivers of lower inpatient hospital costs and greater improvements in health-related quality of life for patients undergoing total shoulder and ream-and-run arthroplasty. J Shoulder Elbow Surg.

[61] Ravi V (2021). Outcome and complications following revision shoulder arthroplasty : a systematic review and meta-analysis. Bone Jt Open.

[62] Anglin C (2001). Loosening performance of cemented glenoid prosthesis design pairs. Clin Biomech (Bristol, Avon).

[63] Welsher A (2019). A comparison of pegged vs. keeled glenoid components regarding functional and radiographic outcomes in anatomic total shoulder arthroplasty: a systematic review and meta-analysis. JSES Open Access.

[64] Scarlat MM, Matsen FA (2001). Observations on retrieved polyethylene glenoid components. J Arthroplasty.

[65] Schmalzried TP (1997). The role of access of joint fluid to bone in periarticular osteolysis. A report of four cases. J Bone Joint Surg Am.

[66] Schmalzried TP, Dorey FJ, McKellop H (1998). The multifactorial nature of polyethylene wear in vivo. J Bone Joint Surg Am.

[67] Rodosky M, Bigliani LU (1994). Surgical treatment of nonconstrained glenoid component failure. Oper Tech Orthop.

[68] Franta AK (2007). The complex characteristics of 282 unsatisfactory shoulder arthroplasties. J Shoulder Elbow Surg.

[69] Martin EJ, Duquin TR, Ehrensberger MT (2017). Reverse total shoulder glenoid baseplate stability with superior glenoid bone loss. J Shoulder Elbow Surg.

[70] Boileau P (2002). Cemented polyethylene versus uncemented metal-backed glenoid components in total shoulder arthroplasty: a prospective, double-blind, randomized study. J Shoulder Elbow Surg.

[71] Papadonikolakis A, Matsen FA (2014). Metal-backed glenoid components have a higher rate of failure and fail by different modes in comparison with all-polyethylene components: a systematic review. J Bone Joint Surg Am.

[72] Ramirez MA, Lu Y, Schaver A (2020). Catastrophic Failure of Reverse Shoulder Arthroplasty from a Broken Screw: A Case Report. JBJS Case Connect.

[73] Kim DM (2020). Do modern designs of metal-backed glenoid components show improved clinical results in total shoulder arthroplasty? A systematic review of the literature. Orthop J Sports Med.

[74] Ladermann A (2019). Glenoid loosening and migration in reverse shoulder arthroplasty. Bone Joint J.

[75] Moroder P, Gerhardt C, Renz N, Trampuz A, Scheibel M (2016). Diagnostik und Management des Endoprotheseninfekts am Schultergelenk. Obere Extremität.

